# Evaluation of a concept to classify anamnesis-related risk of complications and oral diseases in patients attending the clinical course in dental education

**DOI:** 10.1186/s12903-023-03343-x

**Published:** 2023-08-29

**Authors:** Gerhard Schmalz, Lena Brauer, Rainer Haak, Dirk Ziebolz

**Affiliations:** Department of Cariology, Endodontology and Periodontology, University of Leipzig, Liebigstr. 12, 04103 Leipzig, Germany

**Keywords:** Dental education, Risk classification, Medical history, Risk management

## Abstract

**Background:**

Aim of the current study was the assessment of risk classes according to a previously established system to classify the anamnesis-related risk of complications and of oral diseases in a cohort of patients attending the dental student course for dental preventive measures.

**Methods:**

Patients attending the student course between April 2020 and December 2021 were included. To assess the medical history, a specific anamnesis tool was used, which included the classification of different potential anamneis-related risks originating from general diseases, medications or lifestyle factors into a low, moderate or high risk of complications and/or risk of oral diseases. Thereby, the risk of complications was defined as the increaeed probability of harm during dental measures (professional tooth cleaning, invasive dental treatment or any kind of manipulation, which may cause a bacteremia), e.g. infectious endocarditis. On the other hand, the risk of oral diseases was an increaeed probability of development and/or progression of oral diseases like caries, periodontitis or mucosal diseases. Those risk classes were subsequently analyzed under consideration of age and gender.

**Results:**

858 patients, with a mean age of 50.48 ± 20.72 [median: 52.0] years, and a nearly balanced gender distribution (50.8% female and 49.2% male) were included. In the overall cohort, the risk of complications related with dental measures was low in 38.3%, moderate in 42.4% and high in 19.3% of participants. The risk of oral diseases was low in 33.1%, moderate in 37.2% and high in 29.7% of participants. Both, the risk of complications and the risk of oral diseases increased with patients’ age (p < 0.01). Thereby, the risk of complications as well as the risk of oral diseases related to general diseases and medication significantly increased with age (p < 0.01).

**Conclusion:**

Nearly one fifth of patients in dental student couse show a high risk of complications related with dental measures. Morever, nearly one third of those individuals have a high anamnesis-related risk of oral diseases. With increasing age, the amount of patients in high risk classes becomes higher. Future dental education and research should address the high relevance of anamnesis-related risk factors.

**Supplementary Information:**

The online version contains supplementary material available at 10.1186/s12903-023-03343-x.

## Background

For more than three decades, the assessment, evaluation and interpretation of medical histories in dentistry, aiming in the identification and appropriate care of “at-risk” patients in dental practice, is an important research focus [1, 2]. In this context, the terminus “at-risk” is often used for different issues to describe that patients have a medical history, which can lead to problems in dental context. On the one hand, this can mean the probability of harm during or following dental measures, including professional tooth cleaning, invasive dental treatment or any kind of manipulation, which may cause a bacteremia, related with a systemic disease or medication. On the other hand, anamnesis-related factors could also affect the occurrence of oral diseases like caries, periodontitis or mucosal diseases [3]. In both cases, a well-assessed medical history is the starting point of dental care and can be seen as a cornerstone of safe and effective dentistry [4]. Increasing the safety, effectiveness and efficiency is both, the main purpose of personalized medicine and individualized prevention in dental practice [3]. Hence, increasing the safety by avoiding complications, e.g. due to antibiotic prophylaxis to manage the risk of infectious endocarditis, is important [5]. Further, ensuring effective dental care by consideration of systemic conditions affecting oral diseases [6, 7] is of clinical importance. Therefore, a focused, comprehensive and risk-oriented medical history appears crucial.

Especially in dental education, teaching of medical history and consideration of patient-related risk factors is relevant; a previous German study showed that students treated a high number of patients with general diseases during their dental studies [8]. Furthermore, it has been shown that students often overestimate their competency in medical history assessment and interpretation [9], showing a lack in dental education in this field. Although several interventions have been reported, they were primarily performed in context of craniomaxillofacial surgery, especially anticoagulation [10, 11]. Previously, this working group introduced a concept of individualized, risk-oriented prevention, which differentiates risk classes of patients with a low, moderate or high risk in context of preventive dentistry [3]. Thereby, an anamnesis-related risk of complications and a risk of oral diseases can be differentiated and defined. The previously introduced concept describes a risk of complications related with dental measures as an increased likelihood of harm in context of dental therapy, e.g. an infective endocarditis following a bacteremia during professional tooth cleaning [3]. The risk of oral diseases means a higher likelihood to develop oral diseases, including caries, periodontitis, mucosal disease, non-carious tooth decay or oral inflammation as a result of a general disease, medication or lifestyle factors [3]. This classification into low, moderate or high risk can be transferred to all medical history parameters of a patient. This concept was integrated into a teaching concept, which was found to improve the knowledge and to increase the perceived confidence with at-risk patients for dental students [12]. A subsequently developed anamnesis-tool was established and evaluated as well, confirming a benefit of such a medical history teaching in dental education [13]. Although a benefit of risk classification and related medical history tools for dental education appears evident, the practical transfer to dental patients attending the student course remain unclear. However, to improve teaching in medical history for dental students, the real need of the respective patients would be of interest.

Accordingly, this current study applied the upper mentioned risk classification system by using a medical history tool [13] to a cohort of patients attending the student course for preventive measures. The aim of this research was to evaluate the distribution of low, moderate and high risk of complications as well as risk of oral diseases in a mixed group of patients in dental education and to reveal potential associations with age and gender of the patients. It was hypothesized that (I) the amount of patients with a moderate or high risk of complications and/or oral diseases in dental student course would be high and (II) increasing age would be associated with a higher amount of patients with a high anamnesis-related risk of complications and/or oral diseases.

## Methods

### Study design

This current study was a cross-sectional cohort study in patients attending the clinical course in conservative dentistry and periodontology in the Department of Cariology, Endodontology and Periodontology of the Leipzig University. The Ethics Committee of the medical faculty of Leipzig University, Germany reviewed and approved the protocol (No. 487/20-ek). Participating patients were informed verbally and in writing about the study and gave their written informed consent.

### Participants

All patients, who attended the clinical dental education course for preventive measures between April 2020 and December 2021, were consecutively recruited to participate in the current study. Only age of at least 18 years and voluntary participation were inclusion criteria. Any exclusion criteria did not exist.

After information about the current study, patients were asked for their consent to participate. If patients had provided their informed consent, medical history forms of the patients were pseudonymized and copied for data extraction. The resulting data from medical history forms were transferred into a risk classification matrix, which is described below [13].

### Risk classification system and anamnesis tool

Previous studies of this working group described and introduced a risk classification system for medical history information and a respective anamnesis tool [3, 12, 13]. The risk classification is based on a categorization of factors, originating from general diseases, medication and lifestyle into a low, moderate or high risk of complications and/or oral diseases. Accordingly, the system differentiates between a risk of complications, i.e. an increased probability of harm of the patient during dental intervention, and the risk of oral diseases, i.e. the increased likelihood of development or progression of an oral disease [3]. An overview on the different definitions of the risks and respective classes is provided in supplementary Table 1. Based on this classification system, an anamnesis tool was composed, assigning a risk class (low, moderate or high) to each medical history question. For example, a heart valve replacement leads to a high risk of complications (infection risk, requiring antibiotic prophylaxis) and a low risk of oral diseases (no association between valve replacement and oral diseases). This has been applied for all medical history questions as described previously [13]. For analysis, medication groups were built according to their main risk (supplementary Table 2). As relevant lifestyle factors, smoking, alcohol abuse and drug intake/addiction were considered.

### Study flow

After informed consent, the medical history was transferred into the anamnesis tool and a risk classification was performed. Thereby, the overall risk of complications and risk of oral diseases were calculated as depicted in supplementary Table 1. To evaluate the overall risk of a respective patient case, the highest class was considered; thus, if any question was classified as high risk, the overall risk was high, irrespectively whether there was a moderate risk, too. If the highest risk class was moderate, the overall risk was moderate. Only if a question was neither in high nor in moderate class, the overall risk was low. Additionally, the same concept was followed for general disease, medication and lifestyle factors, respectively.

### Statistical analysis

The statistical analysis was performed using SPSS for Windows, Version 24.0 (SPSS Inc., U.S.A.). The frequency of answers in medical history was evaluated as percentage. To compare the different risk classes within risk factors, subgroups with regard to patients´ age were built. Thereby, four similarily sized age groups, which were as homogeneous as possible were statistically built based on the age distribution of the sample. Moreover, a comparison between male and female gender was applied. Categorical data were compared by chi-square test. Nominal data were analyzed with fisher´s exact test. Significance level was set at p < 0.05, whereby two-sided significance testing was executed for all tests.

## Results

### Participants

A cohort of 858 individuals participated in the current evaluation. The mean age of the whole group was 50.48 ± 20.72 (median: 52.0) years, whereby the gender distribution in the sample was nearly balanced (50.8% female and 49.2% male).

### Medical history findings

The results of the overall medical history are shown in Table 1. The most frequent general conditions were hypertonia (31.6%) and allergy (29.5%). Table 2 shows the frequency of medication intake in different medication groups. Therefore, five groups according to medication effects and/or oral side effects were built as shown in supplementary Table 2. Considering those groups, medication with a potential to cause xerostomia were the most frequent ones (monotherapy: 17.5%, combination therapy: 16.2%).

**Table 1:** Results of the general medical history of included indiviuals. Values are given as % (n)

Identifed factors		Amount in participants Participants (n = 858)
General diseases		
Hypertonia		31.6% (271)
Heart valve replacement		0.9% (8)
Arrhythmia		4.8% (41)
Heart failure		0.9% (8)
Stent/Bypass		2.9% (25)
History of heart attack		1.3% (11)
Pacemaker		1.9% (16)
Asthma		7.1% (61)
Chronic obstructive pulmonary disease (COPD)		1.9% (16)
Diabetes mellitus	HbA1c < 7	3.8% (33)
	HbA1c ≤ 7	3.6% (31)
Glaucoma		5.4% (46)
Infectious diseases (Hepatitis, HIV, TBC)		0.7% (6)
Allergy		29.5% (253)
Autoimmune disease	Rheumatoid arthritis	2.6% (22)
	Inflammatory bowel disease	0.1% (1)
Osteoporosis		1,4% (12)
Thyroid disease	Hypothyroidism	13.4% (115)
	Hyperthyroidism	1.3% (11)
Kidney insufficiency		2.9% (25)
Liver disease		2% (17)
Epilepsy		0.9% (8)
Depression		3.5% (30)
Lifestyle factors		
Smoking*	≤ 10 cigarettes/day	9.4% (81)
	> 10 cigarettes/day	6.1% (52)
History of alcohol addiction		0.9% (8)
Drug addiction		1.4% (12)

* The cut–off for the number of cigarettes was defined based on the classification of periodontal diseases [14]

**Table 2:** Medication intake of the patients, categorized into different medication/side effect – groups. Values are given as % (n)

Medication/side effect		Amount in participants Participants (n = 858)
Anticoagulation	Monotherapy	9.9% (85)
	Combination therapy	5.4% (46)
Immunosuppression	Monotherapy	5.2% (45)
	Combination therapy	1.0% (9)
Xerostomia	Monotherapy	17.5% (150)
	Combination therapy	16.2% (139)
Jaw necrosis	Oral	1.4% (12)
	Intra venous	0.8% (7)
Gingival overgrowth		8.0% (69)

### Risk classification

**Fig. 1:**
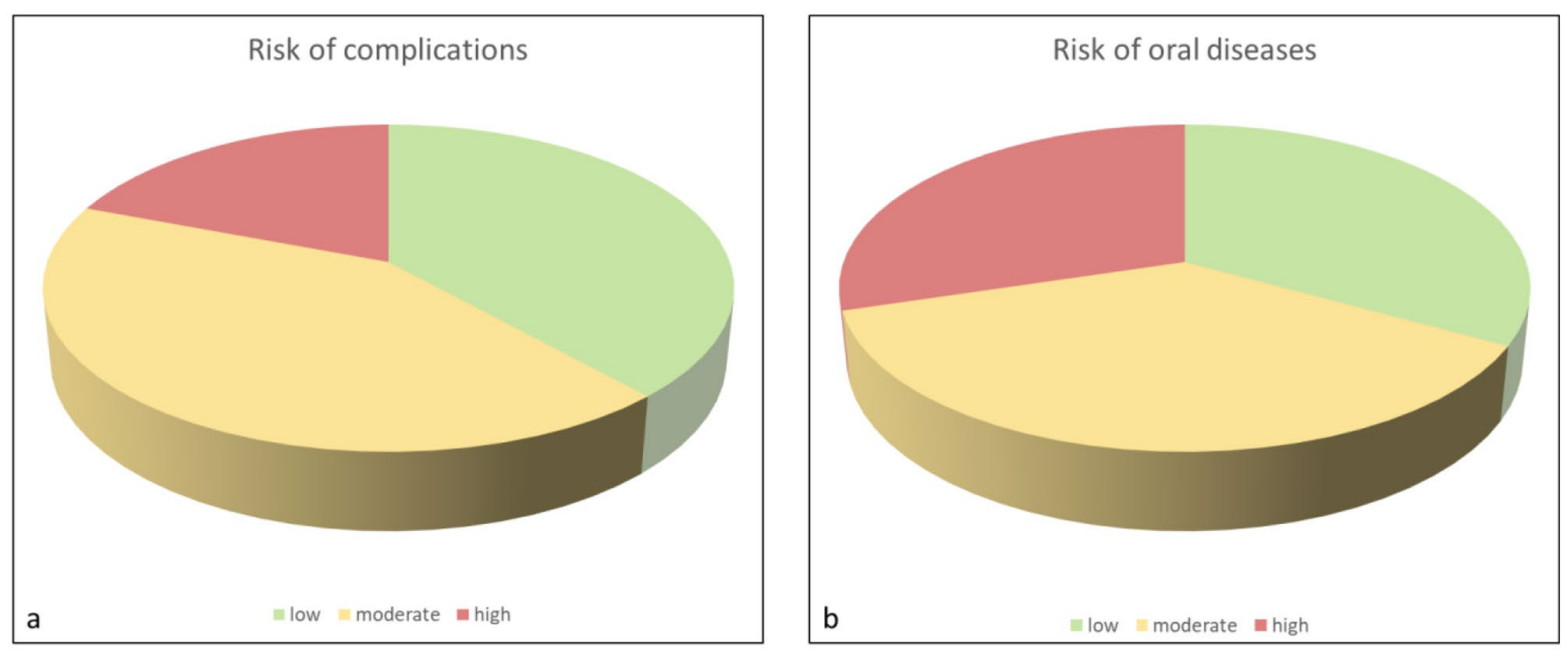
Pie chart of the risk of complications (**a**) and the risk of oral diseases (**b**) in the total cohort

In the overall cohort, the risk of complications was low in 38.3%, moderate in 42.4% and high in 19.3% of participants (Fig. 1a). With regard to the risk of complications, a high risk class was most frequently found because of a general disease (18.9%). The risk of oral diseases was low in 33.1%, moderate in 37.2% and high in 29.7% of participants (Fig. 1b), while it was most frequently affected by the respective medication (21.2%). An overview on the risk classification with regard to general diseases, medication and lifestyle factors is shown in Table 3.

**Table 3:** Risk classification within the three sub-aspects of risk factors in the total cohort (n = 858). Values are given as % (n)

Parameter	Risk class		
	low	moderate	high
***Risk of complications***			
General diseases	40.1% (344)	41% (352)	18.9% (162)
Medication	79.9% (685)	9.3% (80)	10.8% (93)
Lifestyle factors	97.7% (838)	0.9% (8)	1.4% (12)
***Risk of oral diseases***			
General diseases	72.4% (621)	20.7% (178)	6.9% (59)
Medication	64.3% (552)	14.6% (125)	21.1% (181)
Lifestyle factors	83.3% (715)	9.3% (80)	7.4% (63)

### Association between risk classes, age, and gender

Both, the risk of complications and the risk of oral diseases increased with patients’ age (p < 0.01; Table 4). Furthermore, with regard to the risk of oral diseases, a gender difference was detected; men showed significantly less often a moderate risk of oral diseases than women (p = 0.03; Table 5).

**Table 4:** Association between age group and the respective risk class within the risk of complication and the risk of oral diseases. Values are given as % (n)

Parameter	Age group				
	< 28 years (n = 203)	28–51 years (n = 211)	52–69 years (n = 229)	70–89 years (n = 215)	p-value
***Risk of complications***					
Low	68.5% (139)	48.8% (103)	28.8% (66)	9.8% (21)	< 0.01
Moderate	26.1% (53)	37.9% (80)	51.1% (117)	53.0% (114)	
High	5.4% (11)	13.3% (28)	20.1% (46)	37.2% (80)	
***Risk of oral diseases***					
Low	60.6% (123)	35.5% (75)	24.5% (56)	14.0% (30)	< 0.01
Moderate	32.0% (65)	41.7% (88)	41.0% (94)	33.5% (72)	
High	7.4% (15)	22.7% (48)	34.5% (79)	52.6% (113)	

**Table 5:** Association between gender and the respective risk class within the risk of complication and the risk of oral diseases. Values are given as % (n)

Parameter	Gender		
	Male (n = 436)	Female (n = 422)	p-value
***Risk of complications***			
Low	38.9% (164)	37.8% (165)	0.85
Moderate	41.5% (175)	43.3% (189)	
High	19.7% (83)	18.8% (82)	
***Risk of oral diseases***			
Low	35.5% (150)	30.7% (134)	**0.03**
Moderate	32.7% (138)	41.5% (181)	
High	31.8% (134)	27.8% (121)	

**Fig. 2:**
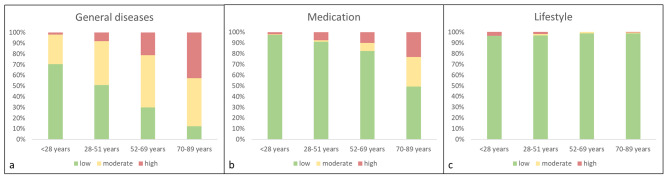
Age dependent differentiation of the risk of complications with regard to the general diseases (**a**), medication (**b**) and lifestyle factors (**c**)

Figure 2a-c show the classification of the risk of complications within the three main groups of risk factors with regard to patient´s age group. It can be seen that the risk of complications related to general diseases and medication significantly increases with age (p < 0.01; Fig. 2a and b). An increased risk of complications regarding lifestyle factors was more evident in younger participants (p = 0.03; Fig. 2c).

**Fig. 3:**
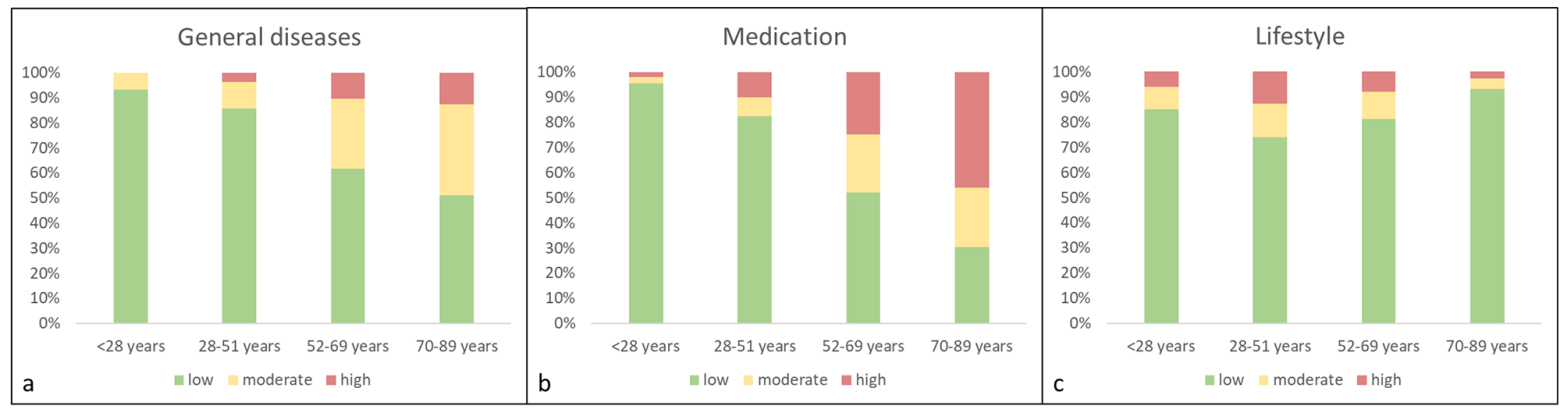
Age dependent differentiation of the risk of oral diseases with regard to the general diseases (**a**), medication (**b**) and lifestyle factors (**c**)

Figure 3a-c illustrate the classification of the risk of oral diseases within the three main groups with regard to patient´s age. Similarly as for the risk of complications, the risk of oral diseases related to general diseases and medication increased significantly with age (p < 0.01; Fig. 3a and b). The risk of oral diseases related to lifestyle factors showed a peak in the middle age group of the cohort (p < 0.01; Fig. 3c).

## Discussion

With an amount of 61.7% moderate or high risk of complications and 66.9% moderate or high risk of oral diseases, risk factors were very common in the cohort. A high risk of complications was mostly caused by general diseases, while a high risk of oral diseases was mostly caused by medication. Altogether, higher age was associated with higher risks. Increasing numbers of general diseases and medication intake appear to result in more patients classified as moderate or high risk of complications and oral diseases, respectively. Accordingly, both hypotheses, which were formulated in the introduction, appear somewhat plausible. However, more research in the field is mandatory; especially larger, representative samples are required to clearly confirm the hypotheses and to draw robust conclusions.

One previous German study, performed by Humbert and co-workers in 2018, evaluated general diseases in a cohort of 511 patients attending a student course [8]. Similarly as in the current study, the previous examination found a high prevalence and number of internal disease in the cohort, whereby hypertension was the most frequent general disease [8]. For the high amount of patients with an allergy, which was found in the current study, no comparable information was found. Until now, a sufficient explanation for the high prevalence of allergy in the current study is missing. However, the previous study did neither consider medication as a factor, nor several other diseases, which were recognized in the current study. Moreover, the risk classification according to the risk-oriented concept of individualized prevention was applied for the first time in the current study. Thereby, general diseases were the main reason for increased risk of complications; this appears plausible, as many diseases, which were assessed in the current study, could lead to complications in dental context. For instance, heart diseases (especially valve replacement) might require antibiotic prophylaxis [5]. Asthma, which was found in 7.1% of participants, is also a relevant risk factor in dental care, where even in context of aerosols and/or applications in preventive care, a risk of ventilatory problems and/or emergencies can occur [15]. Similarly, chronic obstructive pulmonary diseases (COPD) require consideration in dental prevention and care [16]. An insufficiently controlled diabetes mellitus (high HbA1c values) can be related to a risk of infectious complications, potentially requiring an antibiotic prophylaxis [17]. Furthermore, the occurrence of an allergy in nearly 30% is relevant for risk classification in the current study; the risk of anaphylaxis in those patients underline the importance of considering this issue in medical history and to avoid the respective allergens during (preventive) care [18]. Additionally, medications were potential affectors for the risk of complications, whereby especially immunosuppressants, and a related risk of infections, alongside with antiresoprtivants (risk of jaw necrosis) and anticoagulants appear relevant [19–21].

The risk of oral diseases was primarily influenced by the patient’s medication. As shown in Table 2, drugs potentially causing dry mouth were frequently taken by the included individuals. Medication-associated xerostomia is a frequently occurring problem, especially in the elderly [22], what is in line with the current study´s findings. As medication-induced xerostomia increases the caries risk [23], an increased risk of oral diseases is obvious, especially in case of multiple medication. Moreover, medications, which is associated with gingival overgrowth were frequently taken of participants in the current study, resulting in a risk of oral diseases. Those include particularly cyclosporine A, calcium channel blockers and phenytoin [24]. As medication and therefore medication-associated risk increases with age (see Fig. 3b), this appears of particular relevance of dental care and education.

Generally, both the risk of complications as well as of oral diseases increased with age in the current study. As shown in the figures, this is mainly related to the increasing number of general diseases (risk of complications) and medication (risk of oral diseases) with age (see Figs. 2 and 3). It is well reported, that the prevalence of general diseases increases with age, what is caused by ageing as a process of cellular senescence, inflammation, metabolism and oxidative stress [25, 26]. Moreover, the number of drugs increases with age, whereby especially elderly individuals take a variety of medications, with many potential side effects [27]. Against the background of the current demographic change, i.e. the increasing number of patients with higher age and potentially related conditions [28], this appears of particular relevance for dental education in context of risk-oriented prevention, especially under consideration of gerostomatologic curricula and their relevance for dental education and practice. Therefore, as a practical consequence of this current study, teaching concepts require consideration of the respective risks of complications and oral diseases, e.g. using the risk classification concept and anamnesis tool, as introduced previously [12, 13].

Overall, educational research considered the relevance of general diseases in dental context. Especially case-based or problem-oriented learning was shown to be a potential way of teaching clinical medical topics for dental students [29, 30]. On the other hand, it has been shown that internal medical topics are underrepresented in dental studies [31]. In this context, Humbert et al. pick up the approach of integrated education of internal medicine for dental students [8]. This strategy appears plausible, as the current study underlines the need of appropriate education in this field because of the high number of patients with related conditions in dental care settings. Accordingly, developments in medical and dental curricula should be targeted to prepare dental students for their future work in dental practice. This appears especially relevant in context of the well-known associations between oral health, systemic diseases and medication in the elderly [32, 33]. Therefore, dental education needs to focus on those specific individuals [34]. The results of the current study underline the high prevalence of potentially relevant diseases and medication in individuals with higher age. Thus, the anamnesis tool, which was used in the current study, could be used to support dental education in this regard, as it might help to detect and interpret the risks of this patient group faster and easier. While the concept of risk-oriented prevention and the related classification system, which was applied in the current study, could be one component in this context, a superordinate strategy should be elaborated to foster interdisciplinary and interprofessional education in dentistry.

Overall, the importance of the current study for clinical practice requires discussion. The occurrence of patients with a high risk of complications as well as oral disease in all age groups underlines the relevance of the topic. The medical history in dental context is repeatedly discussed to be the significant basis of safe and successful dental therapy [1, 2, 35]. However, this is often challenging in dental practice. For this purpose, the introduced risk classification system and its connection with medical history might be helpful [12, 13]. Thereby, the novelty of the introduced classification system and medical history tool was the connection of general diseases, medication or lifestyle factors directly with a respective risk. The available literature did not develop such an approach, yet. The previous data on the anamnesis tool, which was used in this current study, indicated that the approach to connect medical history information with a risk profile and a related clinical consequence has the potential to increase safety and effectiveness in dental education context [13]. The current data support the relevance to apply the anamnesis tool, as the overall prevalence of at-risk patients was high, especially in elderly individuals. Nonetheless, dental emergencies as relevant complications, which should be covered by evaluating the anamnesis-related risk of complications, repeatedly occur without a respective history, even in dental education [36]. This indicates that further evaluation of the risk groups are needed, especially in prospective perspective on the potential occurrence of complications in the respective patient groups. To improve medical history taking and risk assessment, which was both highlighted previously [37, 38], further progress in this research field is needed. Especially the effect of age on risk class, as mentioned above, could be one piece of the puzzle to improve individualized care in dentistry; future research should evaluate other risk predictors to optimize risk assessment and thus to prevent both, anamnesis-related complications as well as increased development and severity of oral diseases.

With regard to the strengths and limitations of the current study, several further points should be recognized. For the first time, this current study applied the concept of risk classification according to individualized prevention [3] to a cohort of patients attending the student course for dental preventive measures. The sample of more than 850 individuals appears meaningful; however, the assessment was only monocentric and thus not representative for the whole country. Although no complications were reported in the current cohort, this study is not enough to support a practical benefit of using the risk classification system or the anamnesis tool, respectively. However, as shown previously, it appears recommendable to classify the patient-related risks in dental education [13]. With regard to the risk of oral diseases, the oral health situation and oral findings of the patients were not assessed within this project; however, this will be done in a subsequent project. The influence of age was tested by building groups with a similar size in the current study; this might limit the ability to draw conclusions, as this separation is somewhat biased. Moreover, a longitudinal observation would be of interest, to evaluate the development of the respective risks. Additionally, this study did not consider the student´s perspective on usage of the classification system in the educational setting, but this has been evaluated previously [13].

## Conclusion

Within the limitations of this study, nearly one fifth of patients in dental student course show a risk of complications related with dental measures and nearly one third have an anamnesis-related risk of oral diseases. Accordingly, those risks are frequently occurring in dental education context. Thereby, with increasing age, the prevalence of risk factors becomes higher, where the risk of complications is primarily related with more general diseases, while the risk of oral diseases is related with medication. Future dental education should address the high relevance of patient-related risk factors in appropriate teaching strategies.

### Electronic supplementary material

Below is the link to the electronic supplementary material.

Supplementary Material 1

Supplementary Material 2

## Data Availability

The datasets used and/or analysed during the current study are available from the corresponding author on reasonable request. The data are not publically available, because of the psedonymisation and data protection guidelines according to the ethics approval.
