# Supplementary material for: Evaluation of a concept to classify anamnesis-related risk of complications and oral diseases in patients attending the clinical course in dental education
Source: BMC Oral Health. 2023 Aug 29;23:609. doi: 10.1186/s12903-023-03343-x (PMC10466859; doi:10.1186/s12903-023-03343-x)
Supplement: Supplementary file 1 — Supplementary Material 1 [file 12903_2023_3343_MOESM1_ESM.docx]

**Supplementary table 1**: Basis of the risk classification system (mod. after Schmalz and Ziebolz 2020).

| **Class** | | **Systemic diseases, medications, lifestyle factors** | |
| --- | --- | --- | --- |
|  |  | **Risk of complications** | **Risk of oral diseases** |
| **Overall definition** | | **The increaesed probability of harm related with dental measures related with a systemic disease or medication or lifestyle factor of the patient.** | **The increased probability of the development and or progression of an oral disease related with a systemic disease, medication or lifestyle factor of the patient. This risk is independent of the recent oral findings or oral disease history of the patient.** |
| **Low** | Definition | No increased probability of harm related with dental measures | No increased risk of the development and/or progression of oral disease |
|  | Examples | Healthy patient, without general diseases, medication, or harmful lifestyle factors | |
| **Moderate** | Definition | Moderately increased probability of harm related with dental measures | Moderately increased probability for development and/or progression of oral diseases |
|  | Examples | Well controlled COPD, oral bisphosphonate intake, alcohol consumption | Well controlled Diabetes mellitus (HbA1c <7%), Antihypertensive medication (risk of xerostomia), smoking <10 cigarettes/day |
| **High** | Definition | High probability of harm related with dental measures; potentially life-threatening risk | High probability of the development and/or progression of oral diseases |
|  | Examples | Heart valve replacement, immunosuppressive medication, drug consumption | Insufficiently controlled diabetes mellitus (HbA1c >7%), Cyclosporine A medication, smoking >10 cigarettes/day |
