# Supplementary material for: Evaluation of a concept to classify anamnesis-related risk of complications and oral diseases in patients attending the clinical course in dental education
Source: BMC Oral Health. 2023 Aug 29;23:609. doi: 10.1186/s12903-023-03343-x (PMC10466859; doi:10.1186/s12903-023-03343-x)
Supplement: Supplementary file 2 — Supplementary Material 2 [file 12903_2023_3343_MOESM2_ESM.docx]

**Supplementary table 2:** Distribution of the medication subgroups.

| **Problematik** | **Substance class** | **Examples** | **Risk of** | |
| --- | --- | --- | --- | --- |
|  |  |  | **Complications** | **Oral diseases** |
| **Immuno-suppression** | Glukokortikoids | Prednisolone | high | high |
|  | Calcineurine inhibitors | Cyclosporine A, Tacrolimus | high | high |
|  | DNA-Synthesis inhibitors | Mycophenol acid, Azathioprine | high | high |
|  | mTOR-inhibitors | Sirolimus, Everolimus | high | high |
| **Hypo-salivation/**  **Xerostomia** | Antihypertensives | Metoprolole, Ramiprile | low | moderate* |
|  | Antidepresives | Amitryptiline | low | moderate* |
|  | Antihistaminics | Dimetindene, Chlorphenamine | low | moderate* |
|  | Proton pump inhibitor | Omeprazole, Pantoprazole | low | moderate* |
| **Gingival overgrowth** | Calcineurine inhibitors | Cyclosporine A, Tacrolimus | high | high |
|  | Calcium channel blockers | Amlodipine, Nifedipine | low | high |
|  | Antikonvulsives | Phenytoine | low | high |
| **Risk of Jaw necrosis** | Bisphosphonates oral | Zometa, Aclasta | moderate | low |
|  | Bisphosphonates i.v. | Zometa, Aclasta | high | low |
|  | Monoklonal antibodies | Denosumab | high | low |
| **Anti-coagulation** | Thrombocye-aggregation inhibitors | ASS, Clopidogrele | moderate* | low |
|  | Cumarinderivates | Falithrome, Marcumar | moderate** | low |
|  | Direct oral anticoagulative drugs | Dabigatrane, Rivaroxabane | high | low |
|  | Heparine | Heparine | moderate*** | low |

* High in case of combination therapy

** Depending on INR high risk possible

*** Depending on dose high risk possible
